# Climate-related health risks and payer-specific health expenditure growth: empirical evidence from government, social, and out-of-pocket health expenditure in China

**DOI:** 10.3389/fpubh.2026.1921795

**Published:** 2026-08-27

**Authors:** Cunkun Zhang, Qunshan Tao

**Affiliations:** 1 School of Hospital Economics and Management, Anhui University of Chinese Medicine, Hefei, China; 2 Key Laboratory of Philosophy and Social Science of Anhui Province for Data Science and Innovative Development of Chinese Medicine, Hefei, China

**Keywords:** climate-related health risk, health expenditure growth, health financing, IPCC risk framework, structural equation modeling, two-way fixed-effects model

## Abstract

**Background:**

Climate-related health risks may coincide with healthcare needs, service disruptions, and preparedness demands, yet the associated expenditure is distributed across public budgets, collective financing, and households. Evidence rarely shows whether multidimensional climate-risk profiles are associated with expenditure growth across these financing channels. We examined associations between four climate-risk dimensions and real growth in government, social, and out-of-pocket (OOP) health expenditure in China.

**Methods:**

Annual data for 31 provincial-level regions covered 2013–2023; growth models used a balanced panel of 310 province-year observations for 2014–2023. The IPCC framework was operationalized as climate hazard, urban exposure, social vulnerability, and adaptive-capacity deficits. Exploratory and confirmatory factor analyses evaluated the measurement structure. CFA factor scores were entered into two-way fixed-effects models with province and year effects; structural equation modeling (SEM) was supplementary and descriptive.

**Results:**

Measurement analyses were consistent with four related but internally distinguishable dimensions within the study sample. In the primary models, each dimension was positively associated with real growth in all three payer-specific expenditure categories. Positive directions generally persisted under annual *z*-score standardization, 1-year lagging, exclusion of 2020–2022, and log-level outcomes, although magnitudes varied and the social-financing estimates were most sensitive to construct specification. SEM yielded directionally concordant contemporaneous associations but was not used for primary inference.

**Conclusion:**

The findings are consistent with climate-related health risk as a multidimensional financing context whose statistical footprint is visible across multiple payers, while the study does not establish statistically different effects between payers. They support risk-profile surveillance and targeted hypothesis generation for climate-health adaptation, not causal attribution, contribution rules, or province-specific allocation formulas without claims-based and quasi-experimental evidence.

## 1 Introduction

Climate change is increasingly recognized as a health-system challenge, yet its financing consequences remain less clearly mapped. When climate-sensitive illness, service disruption, or preparedness needs arise, the associated expenditure does not enter a single account: public budgets finance public-health and continuity functions, social financing reimburses eligible care under institutional rules, and households pay the residual. This emerging pressure sits alongside established drivers of health-expenditure growth, including population aging, income growth, medical technology, insurance expansion, epidemiological transition, and service prices. Understanding climate-related expenditure therefore requires attention not only to how much spending changes, but also to where that change is recorded.

International frameworks provide the first part of this account by defining the risk that health systems must manage. The IPCC conceptualizes climate risk through interactions among hazard, exposure, and vulnerability and identifies pathways involving injury, chronic disease, infectious-disease transmission, air quality, and food and water security (1). The Lancet Countdown likewise documents risks from heat, extreme weather, food insecurity, reduced labor capacity, and health-system strain (2). WHO guidance consequently integrates climate-risk assessment, continuity of essential services, emergency preparedness, and resource allocation into resilient health-system planning (3), a priority also emphasized in regional Lancet Countdown assessments (4).

Empirical studies establish important links between climate-related exposures, healthcare use, and economic burden. Extreme temperatures are associated with mortality (5), and more intense heatwaves with emergency presentations and healthcare costs (6). In China, heatwaves have been linked to urinary admissions and economic losses (7). Climate change may aggravate many pathogenic diseases (8), while regional assessments document additional pressure on health systems (9). Temperature exposure is also associated with admissions, length of stay, and costs (10), and climate-sensitive events can generate substantial health-related economic burdens (11, 12). Three gaps nevertheless remain: most studies isolate a single exposure, focus on disease-specific or aggregate costs, and do not show how a multidimensional risk profile is reflected across financing sources.

Addressing those gaps requires connecting two layers of the problem. The first is the composition of risk: climate hazard captures the frequency, duration, and intensity of extremes that may coincide with acute illness and service demand (13); urban exposure represents the concentration of people, economic activity, and built space within potential hazard zones (14), where the cooling benefits of green space are also uneven (15); social vulnerability describes demographic and socioeconomic susceptibility (16); and adaptive capacity reflects resources and infrastructure that can prevent, absorb, or reduce harm (17). The second layer is institutional: the same underlying health need may be reflected differently in government, social, and OOP expenditure because eligibility, reimbursement, service access, and public responsibilities differ across payers. This two-layer structure motivates payer-specific analysis without prespecifying a financing hierarchy.

Accordingly, this study asks two linked questions: can provincial climate-related health risk be represented as four empirically distinguishable dimensions, and how are those dimensions associated with real growth in government, social, and OOP health expenditure? The study applies the IPCC hazard–exposure–vulnerability framework while separating vulnerability into social vulnerability and adaptive-capacity deficits. It contributes by bringing multidimensional climate risk into expenditure-growth analysis, tracing its associations across three financing channels, and combining EFA/CFA measurement with two-way fixed-effects estimation. SEM is retained only as supplementary structural-descriptive evidence. Given the provincial observational design, all estimates are interpreted as conditional associations rather than causal effects.

## 2 Materials and methods

### 2.1 Study design, data sources, and sample composition

Annual provincial panel data were used to examine associations between climate-related health risk and payer-specific health expenditure growth in China. The sample comprised 31 provincial-level regions, excluding Hong Kong, Macao, and Taiwan. Source data covered 2013–2023; because annual growth required the preceding year's expenditure, the main models covered 2014–2023 and included a balanced panel of 310 province-year observations.

Government, social, and OOP health expenditure data were obtained mainly from the China Health Statistics Yearbook and provincial statistical publications. Daily meteorological observations came from the China Surface Meteorological Daily Dataset and were used to derive extreme-temperature, extreme-precipitation, and drought indicators. Socioeconomic, urban-construction, demographic, medical-resource, and infrastructure variables were drawn from national and provincial statistical yearbooks and other public sources. Province-year records were harmonized under consistent statistical definitions.

Throughout this article, “payer-specific” is used as analytical shorthand for the three aggregate financing-source categories available in the statistical data, rather than for three mutually exclusive institutions. The aggregate series do not identify individual government programs, insurance schemes, service types, or payment sequences. Accordingly, the institutional mechanisms discussed below are plausible accounting pathways, not observed decompositions of each expenditure category.

### 2.2 Theoretical framework and construction of the indicator system

The analytical framework links a climate-risk production layer to a payer-specific financing-translation layer. Following the IPCC hazard–exposure–vulnerability framework (1), climate-related health risk arises from the conjunction—not equivalence—of climatic stress, people and assets within its reach, and conditions shaping susceptibility and response. Climate hazard captures the frequency, duration, and intensity of extreme events; urban exposure captures population, economic activity, and built-up space that may contact those events. To clarify vulnerability, the study separates social vulnerability—population susceptibility and socioeconomic disadvantage—from adaptive-capacity deficits—limitations in income, education, social protection, green infrastructure, and drainage that may constrain preparation, service continuity, and recovery.

The four dimensions occupy distinct conceptual positions. Hazard is the external stressor and is defined independently of population size or service capacity. Exposure is the potential contact surface, not an indicator of biological or social susceptibility. Social vulnerability concerns sensitivity and unequal ability to avoid harm or obtain timely care, whereas adaptive-capacity deficits concern the institutional, socioeconomic, and infrastructural resources available to reduce exposure, maintain services, and recover. These boundaries prevent population concentration, susceptibility, and response resources from being treated as interchangeable proxies for disadvantage (13–17).

The structure can be viewed as a stressor–contact–susceptibility–buffering architecture. Hazard and exposure describe potential pressure on the health system; vulnerability and adaptive capacity shape whether that pressure becomes illness, disrupted care, delayed treatment, or prolonged recovery. The dimensions may reinforce or offset one another: a highly exposed province may have strong adaptive resources, while moderate climatic stress may still generate substantial strain where susceptibility is high or capacity is weak. The additive model estimates each dimension's conditional association while holding the others constant; it does not assume a deterministic sequence, multiplicative risk formula, or synergistic interaction.

A second distinction concerns the path from risk to recorded expenditure. Risk may first affect health need or service continuity; access, care-seeking, and provider capacity then determine whether need becomes utilization; finally, public responsibilities, eligibility, benefit design, reimbursement, provider payment, and settlement timing determine which account records the expenditure. The same climate-sensitive episode can therefore leave different financial traces across payers, while restricted access or forgone care can suppress recorded expenditure despite high need. The models observe final payer-specific records, not the intervening clinical and institutional transitions.

This separation matters because provinces with similar overall risk can face different underlying problems. Hazard-dominant profiles indicate recurrent climatic stress; exposure-dominant profiles indicate the scale and interdependence of people and built systems at risk; vulnerability-dominant profiles indicate susceptible groups and unequal protective resources; and adaptive-deficit profiles indicate limitations in prevention, continuity, and recovery. Distinct scores therefore provide more information than a single composite index without assuming that one dimension mechanically precedes another.

The payer framework identifies how climate-sensitive needs may appear in financing data. Government expenditure may capture public-good and redistributive functions such as surveillance, emergency response, primary care, medical assistance, continuity infrastructure, and recovery. Social expenditure is an aggregate collective-financing category shaped by insurance participation, pooling, benefit design, deductibles and ceilings, reimbursement, provider payment, cross-regional settlement, and coordination with medical assistance. OOP expenditure comprises direct household payments not reimbursed through government or social financing and depends on care being accessed and recorded. These accounts are complementary rather than mutually exclusive; the provincial models treat them as institutionally distinct aggregate outcomes without claiming to recover the full clinical pathway or contributions of individual programs.

Indicators were selected according to five criteria: theoretical relevance to the assigned construct, a plausible health-system or financing pathway, cross-provincial comparability, temporal continuity, and traceable public sources. Climate hazard included eight indices of temperature extremes, precipitation extremes, and drought persistence. Urban exposure included population, population density, built-up area, urbanization, GDP density, and road density, capturing the scale and connectivity of potential contact rather than susceptibility. Social vulnerability included the shares of children, older adults, registered urban unemployment, and minimum-living-allowance recipients, capturing demographic sensitivity and socioeconomic disadvantage. Adaptive-capacity deficits comprised reverse-coded income, education, social security, green coverage, and drainage-pipeline density, capturing limitations in household resources, collective protection, environmental buffering, and basic urban resilience.

Health technicians and medical-institution beds per 10,000 population appeared in the original specification as response-capacity indicators. Although workforce and beds can support surge response and continuity, they are also produced by prior and current health expenditure and may mediate access and utilization after climate-sensitive events. Including them in a latent predictor of expenditure growth would blur antecedent risk, service capacity, mediating mechanisms, and the outcome. The revised primary model therefore excludes both indicators to reduce mechanical correlation, simultaneity, and construct contamination, while retaining the original composite specification for comparison.

### 2.3 Calculation of climate hazard indicators

Climate-hazard indicators followed established definitions for temperature extremes, precipitation extremes, and drought persistence (18, 19). A fixed 1981–2010 baseline was used to avoid defining extremes from the study period and to meet the 30-year standard for climate normals (20). Definitions are reported in Table 1.

**Table 1:** Coding and definitions of observed variables.

Latent variable	Observed variable	Indicator definition
HAZ	X1	Extreme low temperature: days with daily minimum temperature below the station- and calendar-day-specific historical 10th percentile threshold
HAZ	X2	Cold-spell duration index (CSDI): cumulative days in episodes with daily minimum temperature below the historical 10th percentile for at least 6 consecutive days
HAZ	X3	Extreme high temperature: days with daily maximum temperature above the station- and calendar-day-specific historical 90th percentile threshold
HAZ	X4	Warm-spell duration index (WSDI): cumulative days in episodes with daily maximum temperature above the historical 90th percentile for at least 6 consecutive days
HAZ	X5	Extreme precipitation: days with daily precipitation above the historical 95th percentile of wet-day precipitation
HAZ	X6	Maximum 1-day precipitation (Rx1day, mm)
HAZ	X7	Maximum 5-day precipitation (Rx5day, mm)
HAZ	X8	Maximum consecutive dry days: longest sequence of days with daily precipitation < 1 mm
EXP	X9	Resident population (10,000 persons)
EXP	X10	Population density (persons/km^2^)
EXP	X11	Built-up area (km^2^)
EXP	X12	Urbanization rate (%)
EXP	X13	GDP density (10,000 yuan/km^2^)
EXP	X14	Road density
VUL	X15	Proportion of population aged 0–14 years (%)
VUL	X16	Proportion of population aged 65 years or older (%)
VUL	X17	Registered urban unemployment rate (%)
VUL	X18	Proportion of minimum-living-allowance recipients
AD	X19	Reverse-coded per capita disposable income (yuan)
AD	X20	Reverse-coded average years of education
AD	X21	Reverse-coded social security coverage
AD	X22	Reverse-coded green coverage rate of built-up areas (%)
AD	X23	Reverse-coded drainage-pipeline density (drainage-pipeline length/built-up area)

Per capita disposable income, average years of education, social security coverage, green coverage in built-up areas, and drainage-pipeline density were reverse-coded so that higher values consistently indicated larger adaptive-capacity deficits.

Temperature thresholds were estimated for each station and calendar day using a centered 5-day window during 1981–2010. Extreme low- and high-temperature days were those with daily minimum temperature below the 10th percentile and daily maximum temperature above the 90th percentile, respectively. Cold- and warm-spell duration indices counted days in episodes meeting the relevant threshold for at least six consecutive days.

Precipitation thresholds were based on wet days (≥1 mm). For each station, extreme-precipitation days exceeded the 95th percentile of wet-day precipitation during 1981–2010. Rx1day, Rx5day, and consecutive dry days were the annual maximum 1-day precipitation, maximum 5-day accumulation, and longest run of days with precipitation < 1 mm.

Quality control removed or verified abnormal codes and duplicates, required minimum temperature not to exceed maximum temperature, and excluded negative precipitation. A station-year was retained only when no month had more than three missing days and the year had no more than 15; otherwise it was missing. Daily observations and derived extreme indices were not temporally interpolated.

Station values were averaged to annual provincial indicators using a fixed station sample. Of 370 candidate national reference or basic stations, 338 met baseline-completeness criteria, 321 met study-period availability criteria, and 310 met both (9–14 per province; mean, 10.0 ± 1.4). Provincial means used valid stations from the fixed list and were reported only when at least 80% were available. Stations were not substituted across provinces or added during follow-up, and population weighting was avoided to keep exposure separate from hazard.

### 2.4 Measurement of health expenditure growth rates

The primary outcomes were real annual growth rates of government, social, and OOP health expenditure. Growth rates provide a common measure of annual payer-specific change and reduce, but do not remove, differences in baseline expenditure. They should not be interpreted as direct measures of welfare loss, persistent fiscal stress, or causal financing burden.

Nominal expenditures were deflated to constant 2013 prices using province-year CPI values (previous year = 100) chain-linked to a 2013 = 100 price level. A consistent medical-service price index was unavailable for all provinces and years. If medical-service or pharmaceutical prices rose faster than overall CPI, real growth may be overstated; slower increases would imply understatement. The bias may differ across payers because government procurement, negotiated insurance payments, and household retail prices need not move together. CPI-deflated outcomes are therefore approximate real-growth measures, and future work should assess medical-care, pharmaceutical, or service-specific deflators. Real health expenditure was calculated using Equation (1):


$$\begin{array}{l} \text{RH}{\text{E}}_{i,t,k}=\text{NH}{\text{E}}_{i,t,k}/\left({\text{P}}_{i,t}/\text{1}00\right) \end{array}$$ (1)

Where RHE_i,t,k denotes real health expenditure for financing source k in province i and year t; NHE_i,t,k denotes nominal health expenditure; P_i,t denotes the price index with 2013 as the base year; and k identifies government, social, or OOP health expenditure.

The real annual growth rate for each financing source was then calculated relative to the preceding year using Equation (2):


$$\begin{array}{l} \text{Growt}{\text{h}}_{i,t,k}\text{=}\left[\text{(RH}{\text{E}}_{i,t,k}-\text{RH}{\text{E}}_{i,t-1,k}\text{)/RH}{\text{E}}_{i,t-1,k}\right]\times \text{1}00 \end{array}$$ (2)

Where Growth_*i*,*t*,*k* denotes the real growth rate of health expenditure for financing source k in province *i* and year *t*. Because calculation of the 2014 growth rate required 2013 expenditure as the baseline, the effective outcome period for the primary growth-rate models was 2014–2023.

### 2.5 Variable direction processing and standardization

All climate-risk indicators were direction-adjusted and standardized before measurement-model estimation. The primary analysis used pooled min–max scaling, as shown in Equation (3); robustness analysis used annual cross-provincial z-scores to assess sensitivity to pooled scaling and extreme values.


$$\begin{array}{l} \text{X}\prime_{i,t}=\left[{\text{X}}_{i,t}-\text{min}\left(X\right)\right]/\left[\max\left(X\right)-\text{min}\left(X\right)\right] \end{array}$$ (3)

For adaptive-capacity indicators, for which larger original values represented stronger capacity and therefore lower risk, direction was reversed by maximum-value subtraction, as shown in Equation (4):


$$\begin{array}{l} \text{X}\prime_{i,t}=\left[\text{max}\left(X\right)-{\text{X}}_{i,t}\right]/\left[\max\left(X\right)-\text{min}\left(X\right)\right] \end{array}$$ (4)

After transformation, higher values consistently indicated greater climate-related health risk. Maximum-value subtraction preserved ranks and linear distances while avoiding the instability of reciprocal transformations. Annual *z*-scores represent a province's relative position in that year's cross-provincial distribution, not an absolute risk level. For pooled min–max scaling, min(*X*) and max(*X*) were calculated across all province-year observations in the analysis sample.

### 2.6 Latent-variable measurement and factor-score construction

The empirical workflow followed the research questions. Measurement analysis first tested whether the original combined vulnerability/adaptive-capacity construct could be separated into social vulnerability and adaptive-capacity deficits. A theory-guided EFA examined loading patterns, and CFA assessed the internal coherence of the resulting four-factor structure. Factor scores were generated only after this structure was evaluated.

EFA was conducted within the IPCC framework. Factorability was assessed using the Kaiser–Meyer–Olkin statistic and Bartlett's test. Four factors were fixed for extraction, and Varimax rotation examined whether indicators clustered as climate hazard, urban exposure, social vulnerability, and adaptive-capacity deficits. Because Varimax imposes an orthogonal simple structure, EFA was used to inspect clustering rather than establish substantive independence. Factor retention was theory-guided rather than purely data-driven.

Internal separation was assessed using prespecified criteria: intended loadings ≥0.50, non-target cross-loadings < 0.40, and a gap ≥0.20 between each intended loading and its largest cross-loading (21). Because EFA and CFA used the same repeated province-year sample, these diagnostics provide preliminary internal evidence rather than a comprehensive test of discriminant validity or external validation.

CFA evaluated overall fit, standardized loadings, composite reliability (CR), and average variance extracted (AVE), assessing internal consistency and convergent validity within the sample (21, 22). Fit indices were considered jointly as heuristic diagnostics rather than definitive proof of model validity. Discriminant validity was examined using two complementary procedures. Under the Fornell–Larcker criterion, the square root of each construct's AVE was required to exceed the absolute value of its correlations with every other construct (22). HTMT estimates were evaluated against the prespecified conservative threshold of 0.85 (21). Because both procedures used the same repeated province-year observations as the EFA and CFA, they were interpreted as internal measurement diagnostics rather than independent external validation.

After the revised model showed acceptable fit, reliability, and convergent validity, regression-based CFA scores were generated for the four dimensions in each province-year. These scores became the explanatory variables in the panel analysis. Because two-way fixed-effects coefficients are identified from within-province change after removing common year shocks, they represent conditional within-province associations rather than cross-sectional differences.

### 2.7 Main empirical model: two-way fixed effects

The second analytical stage asked whether changes in the reconstructed risk dimensions were associated with payer-specific expenditure growth within provinces. Two-way fixed-effects models included province and year effects, thereby controlling for time-invariant provincial characteristics and shocks common to all provinces in a given year (23). The four CFA factor scores were entered jointly with observed covariates, and standard errors were clustered at the provincial level. Because exposure, vulnerability, and adaptive resources often evolve gradually, their coefficients are interpreted as conditional within-province associations, not immediate causal effects of 1-year changes. Descriptive within- and between-province variance shares were also calculated for each factor score to assess the amount of variation available for fixed-effects estimation. The primary two-way fixed-effects specification is shown in Equation (5):


$$\begin{array}{r} {\text{Growth}}_{i,t,k}\;=\alpha +\beta_{1}{\text{Hazard}}_{i,t}+\beta_{2}{\text{Exposure}}_{i,t}\\ +\beta_{3}{\text{SocialVulnerability}}_{i,t}+\beta_{4}{\text{AdaptiveDeficit}}_{i,t}\\ +\gamma {\text{Controls}}_{i,t}+\mu_{i}+{\text{λ}}_{t}+\varepsilon_{i,t} \end{array}$$ (5)

Where Growth_*i*,*t*,*k* denotes the real growth rate of health expenditure for financing source *k* in province *i* and year *t*; Hazard_*i*,*t*, Exposure_*i*,*t*, SocialVulnerability_*i*,*t*, and AdaptiveDeficit_*i*,*t* denote the CFA regression factor scores for climate hazard, urban exposure, social vulnerability, and adaptive-capacity deficits, respectively; Controls_*i*,*t* denotes the vector of observed covariates; μ_*i* denotes province fixed effects; λ_*t* denotes year fixed effects; and ε_*i*,*t* denotes the error term.

Covariates were per capita GDP, fiscal self-sufficiency, health insurance participation, province-level CPI, and PM2.5 concentration, representing economic development, local fiscal capacity, insurance coverage, residual general-price variation, and air-pollution-related health risk, respectively. Because CPI also entered the deflation procedure, its coefficient was treated as a nuisance adjustment rather than interpreted structurally.

### 2.8 Supplementary structural equation model

SEM was used only as a supplementary structural-descriptive analysis of contemporaneous relationships between the four risk dimensions and payer-specific expenditure growth. It summarizes directional concordance but lacks province fixed effects, common year shocks, equivalent time-varying controls, and province-clustered inference. SEM therefore cannot replace or strengthen the primary two-way fixed-effects evidence.

### 2.9 Robustness tests

The measurement-specification comparison assessed the sensitivity of payer-specific associations to the original three-factor vs. revised four-factor structure. Four additional analyses tested annual z-score standardization, log real expenditure levels, 1-year-lagged factor scores, and exclusion of 2020–2022, addressing scaling, outcome form, timing, and sample period, respectively. None resolves endogeneity or establishes causality.

### 2.10 Research hypotheses

The hypotheses concern conditional associations, not causal effects or fixed payer rankings. For each dimension, the expected positive direction reflects the net balance of pathways that may raise recorded expenditure—greater need, preparedness, utilization, or recovery spending—and pathways that may attenuate it, including risk reduction, constrained capacity, forgone care, or delayed settlement. Separate payer equations test whether each dimension is statistically visible across financing accounts, not whether one payer necessarily bears a larger effect.

H1: Climate hazard is positively associated with payer-specific real health expenditure growth. More frequent or persistent temperature, precipitation, and drought extremes may coincide with acute morbidity, exacerbation of chronic disease, emergency response, continuity costs, and post-event recovery. These pressures can be recorded as public preparedness or response expenditure, reimbursed medical utilization, direct household payments, or a combination of the three. The expected positive association is therefore common across payers, while its institutional translation remains an empirical question.

H2: Urban exposure is positively associated with payer-specific real health expenditure growth. Concentrated populations, economic activity, transport networks, and built systems may coincide with a larger potentially affected service population and greater complexity in maintaining healthcare continuity. At the same time, urban concentration may improve access, pooling, and economies of scale; the estimated coefficient therefore represents the net association between concentrated exposure and recorded expenditure. Insured utilization may make the association visible in social expenditure, but no payer ordering is prespecified.

H3: Social vulnerability is positively associated with payer-specific real health expenditure growth. Children, older adults, unemployed residents, and low-income populations may have greater biological sensitivity, fewer opportunities to avoid exposure, more chronic-care needs, and less ability to absorb residual medical payments. Public assistance, insurance reimbursement, and household payments may each respond to these conditions. Because poverty and access barriers may also produce delayed or forgone care, a positive coefficient is interpreted as the observed net association rather than proof that all susceptible groups use or pay for more care.

H4: Adaptive-capacity deficits are positively associated with payer-specific real health expenditure growth. Limited income, education, social protection, green infrastructure, and drainage may be consistent with weaker prevention, early response, service continuity, and recovery conditions, under which a comparable climatic stressor may be accompanied by more persistent health-system and financing pressure. Conversely, severe capacity constraints may suppress utilization or recorded claims. The hypothesis therefore concerns the net positive association of weaker adaptive resources with expenditure growth, while the payer channel and the underlying mechanisms remain to be identified with more granular data.

## 3 Results

### 3.1 Exploratory factor analysis of the latent structure of climate-related health risk

The first empirical question was whether the observed indicators recovered the revised four-dimensional risk structure. The KMO statistic was 0.750, and Bartlett's test yielded χ^2^ = 9,741.326 with 253 degrees of freedom (*p* < 0.001), indicating that the correlation matrix was suitable for factor analysis (Table 2).

**Table 2:** KMO and Bartlett's test of sphericity.

Test item	Indicator	Value
KMO test	KMO value	0.750
Bartlett's test of sphericity	Approx. χ^2^	9,741.326
Bartlett's test of sphericity	df	253
Bartlett's test of sphericity	*p*-value	< 0.001

A KMO statistic above 0.70 and a statistically significant Bartlett's test indicate that the observed correlation structure is suitable for exploratory factor analysis.

Table 3 shows a coherent rotated loading pattern. Factor 1 grouped the eight temperature, precipitation, and drought indicators and was labeled climate hazard; Factor 2 grouped population, density, built-up area, urbanization, GDP density, and road density and was labeled urban exposure. These factors represent the climatic stressor and the concentration of people and assets within its potential reach.

**Table 3:** Rotated EFA factor loadings and communalities.

Variable	F1: Hazard	F2: Exposure	F3: Vuln.	F4: Adaptive deficits	Communality
Low-temperature days	0.797	0.043	0.152	0.018	0.660
CSDI	0.726	0.197	0.142	0.241	0.644
High-temperature days	0.739	0.054	0.146	0.246	0.631
WSDI	0.663	0.263	0.152	0.129	0.548
Extreme-precipitation days	0.688	0.088	0.196	0.217	0.567
Rx1day	0.718	0.200	0.127	0.253	0.636
Rx5day	0.701	0.217	0.145	0.235	0.615
Consecutive dry days	0.769	0.128	0.213	0.269	0.725
Resident population	0.128	0.891	0.100	0.024	0.821
Population density	0.148	0.739	0.206	0.039	0.612
Built-up area	0.100	0.901	0.038	0.013	0.823
Urbanization rate	0.141	0.759	0.002	0.014	0.596
GDP density	0.162	0.715	0.268	0.094	0.618
Road density	0.133	0.696	0.234	0.070	0.562
Age 0–14 proportion	0.024	0.206	0.770	0.109	0.648
Age ≥65 proportion	0.218	0.218	0.747	0.232	0.707
Urban unemployment	0.076	0.102	0.716	0.219	0.577
Minimum-living allowance	0.143	0.166	0.706	0.174	0.577
Reverse-coded income	0.078	0.196	0.181	0.855	0.808
Reverse-coded education	0.032	0.129	0.086	0.881	0.801
Reverse-coded social security	0.115	0.251	0.001	0.782	0.688
Reverse-coded green coverage	0.004	0.149	0.010	0.796	0.656
Reverse-coded drainage density	0.187	0.002	0.069	0.847	0.757

Variance contributions were calculated from the rotated sum of squared loadings divided by 23 observed indicators and multiplied by 100%. Four factors were fixed for extraction on the basis of the revised theoretical framework.

Factor 3 grouped the proportions of children and older adults, registered urban unemployment, and minimum-living-allowance receipt and was labeled social vulnerability. Factor 4 grouped reverse-coded income, education, social security, green coverage, and drainage density and was labeled adaptive-capacity deficits. This distinction separates susceptibility from resources for prevention, response, and recovery.

The pattern supports the study's conceptual revision: social vulnerability and adaptive-capacity deficits are related but non-interchangeable dimensions within this measurement specification. Medical-resource indicators were excluded from the revised EFA because their close relationship with expenditure could obscure that distinction.

All indicators met the prespecified simple-structure criteria. Intended loadings ranged from 0.663 to 0.901, the largest non-target cross-loading was 0.269, and the smallest intended-vs.-cross-loading gap was 0.400. Each indicator therefore aligned more strongly with its assigned construct than with alternatives. This is preliminary internal evidence of separation, not comprehensive discriminant validity or external validation.

The four factors jointly explained 66.423% of observed variance: 19.397% for climate hazard, 18.329% for urban exposure, 11.287% for social vulnerability, and 17.409% for adaptive-capacity deficits. The loading pattern and variance decomposition are consistent with four distinguishable elements of a common climate-health risk context.

### 3.2 Confirmatory factor analysis and measurement model fit

The CFA then tested whether the four-factor structure remained coherent under a confirmatory specification. The model showed acceptable fit (Table 4): χ^2^/df = 2.359, CFI = 0.967, TLI = 0.963, RMSEA = 0.062 (90% CI, 0.056–0.074), and SRMR = 0.076. Table 5 reports item loadings and construct-level reliability.

**Table 4:** CFA model-fit indices for the revised four-factor structure.

Fit index	Reference criterion	Model value	Assessment
χ^2^/df	< 3 preferred; < 5 acceptable	2.359	Acceptable
GFI	>0.90	0.946	Acceptable
CFI	≥0.90	0.967	Acceptable
TLI/NNFI	≥0.90	0.963	Acceptable
NFI	≥0.90	0.945	Acceptable
IFI	≥0.90	0.968	Acceptable
RMSEA	≤ 0.08	0.062	Acceptable
RMSEA 90% CI	Upper bound preferably ≤ 0.08	0.056–0.074	Acceptable
SRMR	≤ 0.08	0.076	Acceptable
RMR	< 0.05	0.023	Acceptable
AGFI	>0.90	0.933	Acceptable

**Table 5:** Confirmatory factor analysis of the revised latent variables.

Latent	Item	Std. loading	Unstd. loading	S.E.	*z*-value	*p*	SMC	CR	AVE
HAZ	X1	0.842	1.163	0.273	4.260	< 0.01	0.709	0.929	0.622
HAZ	X2	0.779	1.071	0.257	4.167	< 0.01	0.607	—	—
HAZ	X3	0.731	0.924	0.314	2.943	< 0.01	0.534	—	—
HAZ	X4	0.814	1.034	0.253	4.087	< 0.01	0.663	—	—
HAZ	X5	0.796	0.957	0.296	3.233	< 0.01	0.634	—	—
HAZ	X6	0.749	1.085	0.271	4.004	< 0.01	0.561	—	—
HAZ	X7	0.826	1.154	0.265	4.355	< 0.01	0.682	—	—
HAZ	X8	0.768	0.879	0.226	3.889	< 0.01	0.590	—	—
EXP	X9	0.761	0.956	0.198	4.828	< 0.01	0.579	0.909	0.625
EXP	X10	0.744	0.891	0.268	3.325	< 0.01	0.554	—	—
EXP	X11	0.854	1.003	0.278	3.608	< 0.01	0.729	—	—
EXP	X12	0.808	1.133	0.398	2.847	< 0.01	0.653	—	—
EXP	X13	0.777	0.873	0.192	4.547	< 0.01	0.604	—	—
EXP	X14	0.794	0.986	0.259	3.807	< 0.01	0.630	—	—
VUL	X15	0.785	0.965	0.261	3.697	< 0.01	0.616	0.870	0.626
VUL	X16	0.757	0.927	0.196	4.730	< 0.01	0.573	—	—
VUL	X17	0.798	0.949	0.303	3.132	< 0.01	0.637	—	—
VUL	X18	0.824	0.906	0.203	4.463	< 0.01	0.679	—	—
AD	X19	0.871	1.109	0.325	3.412	< 0.01	0.759	0.912	0.676
AD	X20	0.864	0.979	0.279	3.509	< 0.01	0.746	—	—
AD	X21	0.849	1.247	0.452	2.759	< 0.01	0.721	—	—
AD	X22	0.782	0.871	0.287	3.035	< 0.01	0.612	—	—
AD	X23	0.738	0.887	0.191	4.644	< 0.01	0.545	—	—

HAZ, climate hazard; EXP, urban exposure; VUL, social vulnerability; AD, adaptive-capacity deficits. All corresponding factor loadings were statistically significant at *p* < 0.01. The CFA results provide internal measurement evidence from the same repeated province-year sample used for EFA and should not be interpreted as independent external validation.

Standardized loadings ranged from 0.731 to 0.842 for climate hazard, 0.744 to 0.854 for urban exposure, 0.757 to 0.824 for social vulnerability, and 0.738 to 0.871 for adaptive-capacity deficits. CR values were 0.929, 0.909, 0.870, and 0.912, and AVE values were 0.622, 0.625, 0.626, and 0.676, respectively. All constructs exceeded the conventional CR and AVE thresholds, supporting internal consistency and convergent validity. Tables 6, 7 report the separate Fornell–Larcker and HTMT assessments of discriminant validity, respectively.

**Table 6:** Fornell–Larcker criterion for discriminant validity.

Construct	HAZ	EXP	VUL	AD
HAZ	0.789			
EXP	0.391	0.791		
VUL	0.427	0.417	0.791	
AD	0.395	0.252	0.360	0.822

Bold diagonal entries are the square roots of AVE; lower-triangular entries are latent-variable correlations. The Fornell–Larcker criterion is satisfied when each diagonal value exceeds all correlations involving the same construct. HAZ, climate hazard; EXP, urban exposure; VUL, social vulnerability; AD, adaptive-capacity deficits.

**Table 7:** Heterotrait–monotrait ratio of correlations (HTMT).

Construct	HAZ	EXP	VUL	AD
HAZ	—			
EXP	0.390	—		
VUL	0.430	0.416	—	
AD	0.392	0.249	0.359	—

Lower-triangular entries are HTMT estimates. All values were below the prespecified conservative threshold of 0.85. HAZ, climate hazard; EXP, urban exposure; VUL, social vulnerability; AD, adaptive-capacity deficits.

The Fornell–Larcker results (Table 6) showed that the square roots of AVE ranged from 0.789 to 0.822 and exceeded all corresponding interconstruct correlations, which ranged from 0.252 to 0.427. The HTMT ratios (Table 7) ranged from 0.249 to 0.430 and were all below the prespecified 0.85 threshold. The largest pairwise association was observed between climate hazard and social vulnerability (latent correlation = 0.427; HTMT = 0.430), but both values remained below their respective decision criteria. Taken together, the two diagnostics indicate adequate discriminant validity among the four constructs within the study sample. However, because both were derived from the same repeated province-year observations used for EFA and CFA, they should be interpreted as internal measurement evidence rather than independent external validation.

### 3.3 Comparison of the original three-factor and revised four-factor specifications

The original and revised specifications differ in how they represent the third component of climate-related health risk. The original model combined demographic susceptibility, socioeconomic and infrastructural adaptive-capacity deficits, and medical-resource indicators in one composite factor. The revised model retains hazard and exposure, separates social vulnerability from adaptive-capacity deficits, and excludes medical technicians and beds. This distinction matters because vulnerability concerns susceptibility and barriers to care, adaptive capacity concerns resources for prevention, continuity, and recovery, and medical resources may mediate utilization while also reflecting prior expenditure.

Table 8 compares the two structures using the same sample, outcomes, covariates, fixed effects, and province-clustered standard errors. Hazard and exposure remained positive and significant across all three payers under both specifications. The main difference concerned the third component: the original composite was positively associated with government and OOP growth but not social growth (β = 0.014, *t* = 0.674). After separation, both social vulnerability and adaptive-capacity deficits were positively associated with all three outcomes; the social-expenditure coefficients were 0.101 and 0.093.

**Table 8:** Comparison of the original three-factor and revised four-factor specifications.

Variable	Original Gov.	Original Social	Original OOP	Revised Gov.	Revised Social	Revised OOP
Climate hazard	0.209^***^ (2.870)	0.124^**^ (2.742)	0.114^***^ (4.591)	0.279^***^ (6.628)	0.237^**^ (2.715)	0.250^***^ (4.059)
Urban exposure	0.164^***^ (4.394)	0.107^***^ (3.148)	0.084^***^ (4.009)	0.153^***^ (8.488)	0.219^**^ (2.656)	0.212^***^ (10.526)
Original composite factor	0.065^***^ (3.412)	0.014 (0.674)	0.056^***^ (3.323)	—	—	—
Social vulnerability	—	—	—	0.022^***^ (3.055)	0.101^**^ (2.452)	0.168^***^ (4.723)
Adaptive-capacity deficits	—	—	—	0.222^***^ (3.012)	0.093^**^ (2.606)	0.154^***^ (4.838)
Within *R*^2^	0.477	0.490	0.411	0.509	0.542	0.558
Sample size *N*	310	310	310	310	310	310

In this table compares the original three-factor measurement specification with the revised four-factor specification. The original composite factor included medical-resource indicators, whereas the revised primary model excludes those indicators and separates social vulnerability from adaptive-capacity deficits. Because the comparison simultaneously changes factor separation and indicator composition, it cannot isolate the effect of separation alone. Because the factor scores differ in composition and scale, comparisons concern coefficient direction, statistical significance, and within-model fit rather than numerical coefficient magnitude. ^**^ and ^***^ indicate statistical significance at the 5% and 1% levels, respectively; *t* statistics based on province-clustered standard errors are shown in parentheses.

The contrast is most informative for social financing. This aggregate category reflects collectively financed spending after eligibility, reimbursement, provider-payment, access, and settlement processes. Because the data do not separate individual schemes or components, these mechanisms remain plausible interpretations rather than observed pathways. Combining susceptible populations, weak adaptive resources, and medical capacity can average together pathways that increase reimbursed use, restrict access, or enable claims. The revised models also showed higher unadjusted within *R*^2^ values for government (0.509 vs. 0.477), social (0.542 vs. 0.490), and OOP growth (0.558 vs. 0.411). Because the revised specification contains one additional factor score, part of this increase is mechanical; the values indicate only in-sample fit and do not constitute a formal model-comparison test or establish construct validity or causal superiority; preference for the four-factor structure rests mainly on clearer theoretical boundaries, internal EFA/CFA evidence, and reduced construct contamination.

### 3.4 Main results from two-way fixed-effects models

Table 9 presents the primary two-way fixed-effects estimates. Climate hazard was positively associated with government, social, and OOP expenditure growth (0.279, 0.237, and 0.250, respectively). Urban exposure (0.153, 0.219, and 0.212), social vulnerability (0.022, 0.101, and 0.168), and adaptive-capacity deficits (0.222, 0.093, and 0.154) were also positively associated with all three outcomes. All core coefficients were statistically significant after adjustment for province and year effects and observed covariates. The factor scores showed substantial within-province variation: within-province shares were 59.21% for climate hazard, 57.61% for adaptive-capacity deficits, 51.64% for urban exposure, and 49.60% for social vulnerability. Thus, hazard and adaptive-capacity deficits had larger within- than between-province components, urban exposure was approximately balanced, and social vulnerability was also nearly balanced, with a slightly larger between-province component. These estimates are scale-dependent conditional associations and should not be interpreted as causal effects or directly ranked across factors or outcomes.

**Table 9:** Main results from revised two-way fixed-effects models.

Variable	Government health expenditure growth	Social health expenditure growth	OOP health expenditure growth
Climate hazard	0.279^***^ (6.628)	0.237^**^ (2.715)	0.250^***^ (4.059)
Urban exposure	0.153^***^ (8.488)	0.219^**^ (2.656)	0.212^***^ (10.526)
Social vulnerability	0.022^***^ (3.055)	0.101^**^ (2.452)	0.168^***^ (4.723)
Adaptive-capacity deficits	0.222^***^ (3.012)	0.093^**^ (2.606)	0.154^***^ (4.838)
Per capita GDP per 10,000 yuan	0.005^**^ (2.310)	0.007 (0.517)	0.000 (1.639)
Fiscal self-sufficiency rate	0.001 (0.132)	0.016^**^ (2.386)	0.035^**^ (2.475)
Health insurance participation rate	0.082 (1.671)	0.062 (1.181)	0.170 (0.467)
Consumer price index (previous year = 100)	0.042^***^ (7.654)	0.016^***^ (3.376)	0.098^***^ (5.384)
PM2.5 (μg/m^3^)	0.003 (0.572)	0.057 (1.028)	0.018 (0.231)
Province fixed effects	Yes	Yes	Yes
Year fixed effects	Yes	Yes	Yes
Province-clustered standard errors	Yes	Yes	Yes
Within *R*^2^	0.509	0.542	0.558
Sample size *N*	310	310	310

^**^ and ^***^ indicate statistical significance at the 5% and 1% levels, respectively, based on two-sided t-tests with province-clustered standard errors; *t* statistics are shown in parentheses. The reported *R*^2^ is the within *R*^2^ for the full specification after the fixed-effects transformation and inclusion of the year indicators and covariates; it should not be interpreted as the explanatory contribution of the climate-related risk factors alone.

### 3.5 Robustness tests and supplementary specifications

Table 10 probes sensitivity to temporal ordering and pandemic-period inclusion. With one-year-lagged scores, all four dimensions remained positively and significantly associated with each payer-specific outcome. The same pattern persisted after excluding 2020–2022, indicating that the main findings were not solely driven by contemporaneous measurement or pandemic-era observations. Changes in magnitude and fit nevertheless show specification sensitivity, and neither test establishes causality.

**Table 10:** Robustness tests: one-period lag specification and exclusion of COVID-19 years.

Variable	(1) Lagged Gov.	(2) Lagged Social	(3) Lagged OOP	(4) Excl. COVID Gov.	(5) Excl. COVID Social	(6) Excl. COVID OOP
Climate hazard	0.320^***^ (4.431)	0.229^**^ (2.696)	0.214^***^ (2.968)	0.205^***^ (2.931)	0.253^***^ (4.067)	0.157^***^ (3.928)
Urban exposure	0.252^***^ (6.299)	0.220^**^ (2.744)	0.167^***^ (2.997)	0.176^***^ (3.317)	0.147^***^ (3.811)	0.134^***^ (3.361)
Social vulnerability	0.121^***^ (2.766)	0.114^**^ (2.494)	0.101^***^ (3.374)	0.213^***^ (3.743)	0.217^***^ (3.158)	0.213^***^ (2.893)
Adaptive-capacity deficits	0.147^**^ (2.137)	0.249^**^ (2.625)	0.175^**^ (2.291)	0.201^***^ (3.911)	0.218^***^ (2.913)	0.195^***^ (3.371)
Per capita GDP per 10,000 yuan	0.041 (0.284)	0.000 (0.466)	0.007 (0.106)	0.001^*^ (1.946)	0.005 (1.655)	0.003 (0.518)
Fiscal self-sufficiency	0.061 (0.789)	0.015^**^ (2.446)	0.007^**^ (2.169)	0.002^**^ (2.321)	0.039^***^ (2.784)	0.027 (1.048)
Health insurance participation rate	0.289 (0.534)	0.059 (1.146)	0.021 (0.358)	0.093 (1.055)	0.079 (1.040)	0.066 (1.045)
Consumer price index (previous year = 100)	0.062^***^ (6.843)	0.161^***^ (3.524)	0.049^*^ (1.884)	0.153 (1.104)	0.199 (1.143)	0.047^***^ (3.192)
PM2.5 (μg/m^3^)	0.041^**^ (2.706)	0.010 (1.093)	0.002^**^ (2.201)	0.001^*^(1.964)	0.003 (1.406)	0.005^**^ (2.081)
Province fixed effects	Yes	Yes	Yes	Yes	Yes	Yes
Year fixed effects	Yes	Yes	Yes	Yes	Yes	Yes
Province-clustered standard errors	Yes	Yes	Yes	Yes	Yes	Yes
Within *R*^2^	0.496	0.463	0.476	0.485	0.504	0.491
Sample size *N*	279	279	279	217	217	217

Columns (1)–(3) use one-year-lagged core explanatory variables and omit the 2014 outcome year, yielding 279 observations. Columns (4)–(6) exclude observations from 2020–2022, yielding 217 observations. All models include province and year fixed effects and use province-clustered standard errors. The estimates should not be interpreted as evidence that the pandemic caused differences between specifications. ^*^, ^**^, and ^***^ indicate statistical significance at the 10%, 5%, and 1% levels, respectively; *t* statistics based on province-clustered standard errors are shown in parentheses.

Table 11 replaces pooled min–max scaling with annual cross-provincial z-scores. All four dimensions retained positive associations with each outcome; 11 of 12 coefficients were significant at the 5% or 1% level, and social vulnerability for government expenditure was significant at 10%. Directional consistency suggests that the main positive pattern is not specific to the primary scaling method, although magnitudes are not directly comparable because the scores represent relative annual positions.

**Table 11:** Robustness test using annual *z*-score standardization.

Variable	Government health expenditure growth	Social health expenditure growth	OOP health expenditure growth
Climate hazard (annual z-score)	0.244^***^ (5.495)	0.351^**^ (2.689)	0.302^***^ (3.947)
Urban exposure (annual z-score)	0.159^***^ (5.658)	0.194^***^ (7.125)	0.221^***^ (4.561)
Social vulnerability (annual z-score)	0.185^*^ (2.001)	0.257^***^ (3.355)	0.147^***^ (3.921)
Adaptive-capacity deficits (annual z-score)	0.269^***^ (2.794)	0.237^**^ (2.372)	0.207^***^ (4.173)
Per capita GDP per 10,000 yuan	0.005^***^ (4.102)	0.004^***^ (3.846)	0.001 (1.094)
Consumer price index (previous year = 100)	0.007 (0.898)	0.075^***^ (4.031)	0.013^**^ (2.512)
Health insurance participation rate	0.040 (0.637)	0.027 (0.919)	0.087 (1.102)
Fiscal self-sufficiency rate	0.022^***^ (3.231)	0.142 (1.248)	0.019^***^ (5.384)
PM2.5 (μg/m^3^)	0.018^***^ (2.824)	0.012^**^ (2.426)	0.024 (1.312)
Province fixed effects	Yes	Yes	Yes
Year fixed effects	Yes	Yes	Yes
Province-clustered standard errors	Yes	Yes	Yes
Within R^2^	0.501	0.486	0.503
Sample size N	310	310	310

Observed indicators were standardized within each year using z-scores across provinces before factor-score construction. The resulting estimates represent associations with a province's relative position in the annual cross-provincial distribution. All models include province and year fixed effects and province-clustered standard errors. ^*^, ^**^, and ^***^ indicate statistical significance at the 10%, 5%, and 1% levels, respectively; *t* statistics based on province-clustered standard errors are shown in parentheses.

Table 12 uses log real expenditure levels instead of annual growth rates, retaining the same 2014–2023 estimation sample. All 12 core coefficients remained positive and significant, indicating that the central pattern is not confined to the growth-rate definition. Because level models are more sensitive to province size, baseline expenditure, trends, and common shocks, they are supplementary and do not replace the growth specification.

**Table 12:** Supplementary two-way fixed-effects models using log real health expenditure levels.

Variable	Log real government health expenditure	Log real social health expenditure	Log real OOP health expenditure
Climate hazard	0.301^***^ (3.820)	0.318^***^ (3.917)	0.476^**^ (2.405)
Urban exposure	0.252^***^ (4.555)	0.205^**^ (2.547)	0.279^**^ (2.728)
Social vulnerability	0.137^**^ (2.650)	0.231^***^ (2.953)	0.244^***^ (4.740)
Adaptive-capacity deficits	0.134^***^ (6.734)	0.034^***^ (2.794)	0.431^***^ (7.177)
Per capita GDP per 10,000 yuan	0.070 (0.428)	0.000 (1.042)	0.188 (0.596)
Consumer price index (previous year = 100)	0.016^***^ (5.162)	0.006^***^ (4.312)	0.110^***^ (8.786)
Health insurance participation rate	0.263 (0.462)	0.010 (0.241)	0.022 (0.062)
Fiscal self-sufficiency rate	0.039^***^ (7.927)	0.019^***^ (3.987)	0.023^***^ (8.538)
PM2.5 (μg/m^3^)	0.019^**^ (2.310)	0.023^***^ (3.131)	0.047^***^ (2.910)
Province fixed effects	Yes	Yes	Yes
Year fixed effects	Yes	Yes	Yes
Province-clustered standard errors	Yes	Yes	Yes
Within *R*^2^	0.521	0.497	0.535
Sample size *N*	310	310	310

The dependent variables are the natural logarithms of real health expenditure levels deflated to constant 2013 prices. Because expenditure levels are more sensitive to province size and baseline scale, these models provide supplementary evidence and do not replace the growth-rate specification. ^**^ and ^***^ indicate statistical significance at the 5% and 1% levels, respectively; *t* statistics based on province-clustered standard errors are shown in parentheses.

### 3.6 Supplementary structural equation modeling results

SEM was retained only to assess directional concordance in contemporaneous associations, not as a competing inferential model. Because it omits province and year fixed effects, equivalent time-varying controls, and province-clustered inference, Table 13 compares only coefficient signs and broad patterns; SEM magnitudes are not directly comparable with two-way fixed-effects estimates. The corresponding supplementary SEM association coefficients are reported in Table 14.

**Table 13:** Directional comparison of primary fixed-effects and supplementary SEM estimates.

Association	Fixed-effects coefficient	SEM standardized coefficient	Directional comparison
Climate hazard–Government	0.279^***^	0.357^***^	Positive in both
Climate hazard–Social	0.237^**^	0.253^***^	Positive in both
Climate hazard–OOP	0.250^***^	0.426^**^	Positive in both
Urban exposure–Government	0.153^***^	0.218^**^	Positive in both
Urban exposure–Social	0.219^**^	0.302^**^	Positive in both
Urban exposure–OOP	0.212^***^	0.359^***^	Positive in both
Social vulnerability–Government	0.022^***^	0.377^**^	Positive in both
Social vulnerability–Social	0.101^**^	0.330^**^	Positive in both
Social vulnerability–OOP	0.168^***^	0.204^**^	Positive in both
Adaptive-capacity deficits–Government	0.222^***^	0.342^**^	Positive in both
Adaptive-capacity deficits–Social	0.093^**^	0.203^**^	Positive in both
Adaptive-capacity deficits–OOP	0.154^***^	0.337^**^	Positive in both

^**^ and ^***^ denote *p* < 0.05 and *p* < 0.01, respectively. Fixed-effects significance is based on two-sided *t*-tests with province-clustered standard errors; SEM significance is based on two-sided *z*-tests.

**Table 14:** Supplementary SEM association coefficients.

Association	Unstd. coeff.	S.E.	*z*-value	*p*	Std. coeff.	Evidence
Hazard–Gov.	0.107	0.0410	2.610	0.009	0.357	Positive
Exposure–Gov.	0.212	0.1016	2.087	0.037	0.218	Positive
Vuln.–Gov.	0.101	0.0423	2.388	0.017	0.377	Positive
Adaptive def.–Gov.	0.218	0.0857	2.544	0.011	0.342	Positive
Hazard–Social	0.104	0.0392	2.653	0.008	0.253	Positive
Exposure–Social	0.302	0.1202	2.512	0.012	0.302	Positive
Vuln.–Social	0.124	0.0533	2.326	0.020	0.330	Positive
Adaptive def.–Social	0.223	0.0917	2.432	0.015	0.203	Positive
Hazard–OOP	0.210	0.0916	2.290	0.022	0.426	Positive
Exposure–OOP	0.212	0.0737	2.877	0.004	0.359	Positive
Vuln.–OOP	0.330	0.1654	1.995	0.046	0.204	Positive
Adaptive def.–OOP	0.311	0.1252	2.484	0.013	0.337	Positive

Unstd. coeff., unstandardized coefficient; Std. coeff., standardized coefficient; S.E., standard error. The *z*-value equals the unstandardized coefficient divided by its standard error, and *p* values are two-sided.

All 12 SEM associations were positive and significant. The largest standardized coefficient within each risk dimension was observed for OOP growth for climate hazard (0.426) and urban exposure (0.359), and for government growth for social vulnerability (0.377) and adaptive-capacity deficits (0.342). These results are directionally concordant with the primary models but remain descriptive and may reflect between-province differences, common time trends, or omitted confounding.

## 4 Discussion

### 4.1 Main findings

The findings support a four-part account of climate-related health risk and financing. Climate hazard, urban exposure, social vulnerability, and adaptive-capacity deficits were related yet empirically distinguishable within the sample. Within-province increases in each dimension were positively associated with real growth in government, social, and OOP expenditure after accounting for province effects, common year shocks, and observed covariates. The same risk context was therefore visible across multiple payers, consistent with institutional translation rather than exclusive assignment to one financing source. However, some estimates—especially for social financing—were sensitive to measurement construction, underscoring their conditional and specification-dependent nature.

The main contribution is theoretical and institutional rather than a ranking of coefficients. The four factors locate different parts of the risk architecture—stressor, contact, susceptibility, and buffering resources—while the three outcomes locate different financing functions—public provision, rule-filtered collective financing, and household residual payment. Linking these architectures helps explain why similar climate-health pressures may be recorded differently across provinces and years. Because factor scales and payer accounting processes differ, coefficient magnitudes should not be treated as directly comparable effect sizes or as evidence of mechanically shifting responsibility. Conceptually, these estimates describe accounting incidence—where expenditure is recorded—rather than ultimate economic incidence or total welfare burden.

Separating social vulnerability from adaptive-capacity deficits improves interpretation, not merely fit. Social vulnerability concerns who is more likely to experience harm or barriers to care (16), whereas adaptive capacity concerns whether households, institutions, and infrastructure can prevent escalation, maintain services, and recover (17). A composite factor can obscure provinces with high susceptibility but strong infrastructure, or weak capacity but lower population vulnerability. Table 8 is consistent with this distinction: the original composite was not associated with social financing, whereas the revised model identified separate positive associations for vulnerability and adaptive deficits. Because the revised specification also removed medical-resource indicators, the comparison cannot isolate the effect of factor separation alone; its advantage is greater theoretical and institutional resolution.

Successive Lancet Countdown assessments document a widening climate-health agenda: the 2021 report emphasized escalating risks involving heat, infectious disease, food security, and health-system capacity (24); the 2023 and 2024 reports called for health-centered responses as harms became harder to reverse (25, 26); and the 2025 report highlighted health gains from accelerated action (27). Evidence from small island developing states illustrates the interaction of high exposure and limited capacity (28), while China-specific assessments identify substantial risks and policy opportunities (29–32). This study adds a financing lens by tracing how distinct risk components appear across institutions and households.

Recent Chinese evidence supports this lens. Provincial climate risk has been associated with insurance demand, particularly health insurance (33); extreme temperatures have been associated with household OOP medical expenditure (34); and climate conditions have been linked to medical-service supply and spatial spillovers (35). Heatwave-attributable injury admissions and costs vary geographically (36), and climate-related health inequality differs across insurance tiers among middle-aged and older adults (37). Together, these findings indicate that exposure alone cannot explain climate-health expenditure; institutional design, vulnerability, and provincial context shape where pressure becomes visible.

Payer-specific estimates do not formally establish differences across payers. The outcomes have different distributions, accounting rules, and potentially correlated disturbances, and no cross-equation Wald tests were conducted. Differences in coefficient size therefore inform institutional interpretation and hypotheses only. Stronger heterogeneity claims require common scaling, joint estimation, and explicit equality tests across the three equations.

### 4.2 Climate hazard and payer-specific expenditure growth

Climate hazard is the framework's most immediate external component, capturing the occurrence and persistence of temperature, precipitation, and drought extremes. Its positive association with all three payer categories suggests that acute climatic stress can appear in multiple financing accounts. Expenditure may occur before events through surveillance and preparedness, during events through emergency and clinical services, or afterward through follow-up care, restoration, and delayed settlement; these functions are central to climate-resilient health-system planning (3). Annual data combine these time horizons, so the coefficient is a broad contemporaneous risk signal rather than a diagnosis- or event-specific payment effect.

This interpretation is biologically, operationally, and economically plausible. Non-optimal temperature is linked to mortality (5), admissions, length of stay, and healthcare costs (10), while climate-sensitive events can impose substantial economic burdens (11). Attribution studies quantify warm-season mortality related to human-induced climate change (38), and evidence from Adelaide links changing thermal conditions to emergency presentations and costs (39). Hazards may also disrupt transport, staffing, supply chains, electricity, referral pathways, and chronic-care continuity. These channels explain why a hazard signal may appear across financing sources, although the provincial models cannot identify the diagnoses, services, or payment sequences involved.

Severe heat illustrates why a common positive direction need not imply a uniform response. The 2022 European summer produced substantial heat-related mortality (40); multicountry evidence demonstrates substantial geographic variation in temperature-attributable mortality (41); and global models identify cause-specific risks across non-optimal temperatures (42). Temperature-attributable respiratory hospitalization costs may rise under future climates (43), while high-mortality heat seasons are projected to become more frequent (44). Associations may therefore vary with event intensity, duration, baseline health, adaptation, access, and payment rules. The annual provincial design averages over this heterogeneity and is not an event-level dose–response analysis.

The government and social-financing associations are also compatible with climate-resilient health-system requirements. Preparedness, continuity of care, resilient supply chains, adaptive resource allocation, and low-carbon resilience require institutional action (45), while WHO guidance emphasizes governance, financing, workforce, information, service delivery, and infrastructure (46). Government expenditure may capture public goods and readiness even without a reimbursable encounter, whereas social expenditure may record covered utilization after eligibility and settlement. These differences help explain why hazard is visible in both accounts without implying identical mechanisms or a stable payer ranking.

### 4.3 Urban exposure and payer-specific expenditure growth

Hazard describes climatic stress; urban exposure describes the scale and interdependence of people, economic activity, and built systems within its reach. Urban heat-vulnerability research identifies population concentration and built form as central components of urban heat risk (14). Urban exposure was positively associated with government, social, and OOP growth, with positive signs generally retained in robustness analyses. In dense urban settings, climate-sensitive events may affect larger service populations, create simultaneous demand, and disrupt transport, power, referral, and supply systems. Urban concentration can also improve access, provider density, fiscal capacity, and economies of scale. The coefficient therefore represents the net within-province association of concentrated exposure with recorded expenditure, not evidence that urbanization is intrinsically harmful.

The exposure score combines population, density, built-up area, urbanization, GDP density, and road density, capturing the size, concentration, and connectivity of the potential demand surface rather than susceptibility; this distinction is consistent with urban heat-vulnerability frameworks (14). Conditional on hazard and the other factors, greater exposure may coincide with more outpatient visits, emergency presentations, hospitalizations, public-health operations, and household payments because more people and interdependent services are simultaneously at risk. Strong infrastructure or spatially targeted adaptation may attenuate this relationship. Exposure should therefore be interpreted jointly with, but not substituted for, social vulnerability and adaptive capacity.

Urban heat-vulnerability research supports treating population concentration and the built environment as exposure components (14), while green-space cooling is substantial but unequally distributed (15). The positive social-expenditure association is consistent with concentrated insured utilization and greater claims visibility in urban settings, although provider availability, benefit design, and faster settlement are alternative explanations. Neither SEM nor the provincial panel establishes social financing as the dominant urban channel; the findings instead indicate that financial expression depends on access, public provision, insurance rules, and local adaptive resources.

### 4.4 Social vulnerability, adaptive-capacity deficits, and payer-specific expenditure growth

Social vulnerability and adaptive capacity answer different questions in the climate-health pathway. Social vulnerability identifies populations more susceptible to harm or barriers to care (16), whereas adaptive capacity concerns the resources and institutions available to prevent escalation, maintain services, and recover (17). The revised model distinguishes social vulnerability—age structure, unemployment, and minimum-living-allowance receipt—from adaptive-capacity deficits in income, education, social protection, green infrastructure, and drainage. This separates a distributive problem concerning susceptible populations from a system-capacity problem concerning the resources and institutions that buffer risk.

Social vulnerability was positively associated with all three outcomes. Larger shares of children, older adults, unemployed residents, and low-income populations may indicate greater biological sensitivity, chronic-care dependence, limited exposure avoidance, and barriers to timely care, consistent with the social-vulnerability framework (16). Chinese evidence further links extreme temperatures to household OOP medical expenditure (34), while multi-tier insurance arrangements are associated with climate-related health inequality among middle-aged and older adults (37). These conditions can appear in public assistance, reimbursed use, or household payment. Yet low income and access barriers may also delay or suppress care, reducing recorded expenditure despite unmet need. The positive coefficients therefore represent net population-level associations and signals for distributional investigation, not proof that every vulnerable group used or paid for more services.

Adaptive-capacity deficits were also positively associated with all three outcomes. Limited household resources, education, social protection, green coverage, and drainage may weaken prevention, risk communication, service continuity, and recovery, allowing similar climatic stress to generate more severe or persistent pressure (17). Climate-resilient health-system frameworks likewise identify continuity, resilient infrastructure, and adaptive resource allocation as core capacities (45, 46). The larger government coefficient in the primary model is compatible with public responsibilities for emergency response, primary care, surveillance, assistance, and resilient infrastructure, but these channels were not observed separately and payer coefficients were not formally compared. Because capacity constraints may also suppress utilization and claims, the estimate reflects the net result of need, access, response, and accounting processes.

The distinction changes policy interpretation. A vulnerability-dominant profile raises questions of targeting, outreach, chronic-care continuity, and financial protection for susceptible groups (16). An adaptive-deficit profile raises questions of infrastructure, institutional readiness, social protection, and the ability to absorb and recover from shocks (17). Combining the two can conceal divergent trajectories, such as improving infrastructure alongside population aging or declining household resources despite stable hazard.

The model comparison illustrates how measurement structure shapes institutional interpretation. In the original three-factor model, the combined vulnerability–adaptive-capacity factor was associated with government and OOP growth but not social growth. The composite mixed demographic susceptibility, socioeconomic and infrastructural deficits, and medical capacity—components that may increase covered need, restrict access, prolong pressure, or enable claims in different directions. After vulnerability and adaptive deficits were separated and medical-resource indicators removed, both dimensions were positively associated with social growth. The revised specification therefore revealed two distinct signals obscured by the broader composite, although factor separation and indicator removal occurred together and their separate contributions cannot be identified.

Medical technicians and beds were excluded because they may be adaptation inputs, mediators of access and surge capacity, moderators of the hazard–utilization relationship, and outcomes of prior health investment. Evidence that climate conditions can affect medical-service supply and generate spatial spillovers reinforces the need to model service capacity separately from antecedent risk (35). Exclusion reduces mechanical overlap with expenditure and sharpens the distinction between pre-existing risk and financing response. Future studies should model medical resources separately and test whether they buffer morbidity, convert unmet need into utilization, or transmit prior investment into current expenditure.

### 4.5 Interpretation across payer-specific expenditure categories

Taken together, the payer-specific results form an institutional accounting map of aggregate financing sources. Government expenditure may reflect public-good, redistributive, preparedness, and continuity functions, consistent with climate-resilient health-system guidance (46). Social expenditure may reflect rule-filtered collective financing, including covered utilization, and climate risk has been associated with insurance demand in China (33). OOP expenditure records direct household payments not otherwise reimbursed, conditional on care being accessed and recorded; extreme-temperature evidence from China documents this household-payment channel (34). One climate-sensitive episode may appear in several accounts—for example, through public response, an insured encounter, and a household copayment—and fiscal booking or insurer settlement may not coincide with service use.

This perspective explains why the outcomes are non-exclusive and why coefficient differences do not define a financing hierarchy. A common risk profile can generate different financial observations across provinces and years, while weak access may leave substantial need under-recorded in every account. The aggregate accounts also omit indirect losses and unmet need that never becomes recorded care. Stronger interpretation requires linking factor scores to event timing, diagnoses, utilization, provider charges, reimbursement shares, settlement delays, government transfers, and household payments. Formal payer comparisons also require joint estimation and cross-equation tests.

### 4.6 Cross-provincial heterogeneity and interpretation

Provincial heterogeneity means that the same broad association can imply different operational priorities. Climate regimes and medical-service supply may exhibit spatial spillovers (35), while heatwave-attributable injury burden and costs vary geographically (36). Insurance demand and financial protection also differ across climate-risk and insurance contexts (33, 37). Urban form, age structure, income, fiscal capacity, healthcare access, green infrastructure, and drainage may further condition both the conversion of risk into service pressure and the translation of services into payer accounts. An exposure-dominant province may warrant assessment of surge capacity and system continuity; a vulnerability-dominant province may warrant assessment of outreach, medical assistance, and residual-cost protection; and a capacity-deficit province may warrant assessment of sustained infrastructure, social protection, and primary-care investment. Provinces with compound profiles may require coordinated assessment across payers and across the pre-event, response, and recovery phases.

These are risk-profile interpretations, not rankings of named provinces or validated allocation rules. Evidence links climate risk to insurance demand (33), extreme temperatures to household medical expenditure (34), climate conditions to service-supply spillovers (35), heatwaves to geographically varying injury costs (36), and insurance tiers to unequal climate-related health outcomes (37). Future work should publish uncertainty-quantified provincial profiles, test temporal stability, and link them to claims, utilization, fiscal accounts, medical-assistance records, and household surveys. Such evidence could support transparent, locally calibrated preparedness while avoiding ecological labeling.

## 5 Conclusions and policy implications

### 5.1 Conclusions

Using data from 31 provincial-level regions for 2013–2023, with growth models covering 2014–2023, this study linked the composition of climate-related health risk to its translation across government, social, and household financing. Climate hazard, urban exposure, social vulnerability, and adaptive-capacity deficits showed internally distinguishable measurement patterns and were each positively associated with real growth in all three payer-specific expenditure categories. The revised model separated population susceptibility from buffering resources and excluded medical-resource indicators to reduce conceptual overlap with expenditure. Two-way fixed effects provide the primary evidence; SEM is supplementary and descriptive, and robustness analyses demonstrate directional persistence rather than causal identification.

Climate-related health financing cannot be inferred from hazard alone. Its financial trace may depend on the stressor, scale of potential contact, population susceptibility, buffering resources, and institutional rules that translate service use into public, collective, or household accounts. The findings do not show that climate risk mechanically causes expenditure growth, that apparent coefficient differences are significant across payers, or that one payer consistently bears the largest burden. They support multidimensional surveillance, payer-specific hypothesis generation, and preparedness planning, but not contribution rates, reimbursement rules, transfer formulas, or province-specific budgets without causal, claims-based, and more granular evidence.

### 5.2 Policy implications

The policy logic follows a risk-profile × financing-lever × time-horizon framework. The risk-profile component follows the IPCC's interacting hazard, exposure, and vulnerability framework (1), while the financing and time-horizon components are consistent with WHO guidance on preparedness, continuity, response, and recovery (46). Authorities could first identify whether hazard, exposure, social vulnerability, adaptive-capacity deficits, or a compound configuration dominates locally; then determine which payer or institution holds the relevant operational lever; and distinguish preparedness, acute response, and recovery. Government budgets, social financing, and household OOP protection can thus be treated as complementary instruments with shared monitoring but different responsibilities. The recommendations below are precautionary applications of observed associations, not estimated policy effects or allocation formulas.

#### 5.2.1 Government fiscal budgets and public-health preparedness

Government budgeting could use the four dimensions to support a trigger-based preparedness cycle rather than a single allocation score. Before events, high-hazard areas may need contingency reserves, early-warning links, pre-positioned supplies, protected essential-service funding, and predefined activation thresholds, consistent with climate-resilient health-system planning (46). Highly exposed urban areas may need surge plans, referral protocols, resilient power and supply systems, and real-time demand monitoring; urban-vulnerability and resilient-healthcare research support attention to these interconnected systems (14, 45). During and after events, socially vulnerable areas may require medical assistance, outreach, transport, cooling, and medicine continuity (16). Areas with large adaptive deficits may require targeted transfers for drainage, green or cooling infrastructure, resilient primary care, and workforce support (15, 17). Separating ex ante resilience investment, event-activated response, and recovery expenditure—and linking each to release triggers and performance indicators—would improve evaluation.

#### 5.2.2 Social medical insurance fund resilience

Because social health expenditure is an aggregate category, the insurance implications below concern one plausible component and require claims-based validation. Climate risk has been associated with insurance demand in China (33), while insurance-tier differences are associated with climate-related health inequality (37). These findings support building climate-sensitive claims intelligence and stress-testing systems before altering contribution or benefit rules. Surveillance could identify climate-sensitive episodes and track service type, diagnosis, age, insurance type, reimbursement share, provider, location, cross-regional settlement, and settlement lag, distinguishing utilization pressure from accounting timing. Stress tests could examine fund cash flow and household cost shifting under different pooling levels, benefits, deductibles and ceilings, provider payment, medical-assistance coordination, and regional risk concentration. Temporary risk sharing, accelerated settlement, reserve use, or benefit adjustment should be considered only when linked claims show reproducible pressure, inequity, and credible cost shifting to OOP payment.

#### 5.2.3 Household out-of-pocket financial protection

Household protection should address residual financial and access consequences after insurance and public response. Extreme temperatures have been associated with household OOP medical expenditure in China (34), and insurance-tier differences are associated with climate-related health inequality among middle-aged and older adults (37). Monitoring could therefore cover event-linked OOP payments, catastrophic expenditure, medicine interruption, delayed or forgone care, and distress financing among susceptible groups. Where rules permit, basic insurance, medical assistance, temporary assistance, and disaster relief could be coordinated through proactive identification, simplified documentation, rapid reimbursement, and continuity of essential medicines. Financial measures may need cooling, shelter, transport, outreach, telehealth, and referral support because lower copayments do not help households unable to reach care; resilient-healthcare frameworks emphasize continuity and access during climate shocks (45, 46). Temporary relief or coverage expansion requires pilot testing, time limits, distributional evaluation, and redesign when benefits are not demonstrated.

#### 5.2.4 Cross-provincial implementation by risk profile

Cross-provincial implementation could be organized around measured risk configurations, institutional readiness, and uncertainty rather than a single ranking. Repeated profiling is consistent with the IPCC's interacting risk framework (1) and WHO's adaptive approach to climate-resilient health-system planning (46). Each profile below identifies a dominant risk signal, financing functions that may warrant attention, and follow-up information needs. Profiles can overlap and change as climate, population, infrastructure, and policy evolve. Operational use therefore requires repeated profiling, uncertainty intervals, transparent indicators, and periodic review rather than permanent labels.

High climate-hazard profile. Potential monitoring and preparedness priorities include integrated early warning, contingency reserves, pre-positioned medicines and supplies, mobile or referral capacity, and event-linked monitoring of utilization and OOP payments. These priorities are consistent with evidence on heat-related health risks and climate-resilient preparedness (13, 46).

High urban-exposure profile. Potential monitoring and preparedness priorities include claims-based early warning, surge-capacity and cross-regional settlement planning, and climate-resilient power, transport, medicine, and supply chains. Urban-vulnerability research supports prioritizing densely exposed systems (14), while resilient-healthcare literature emphasizes continuity of infrastructure and supply chains (45).

High social-vulnerability profile. Potential monitoring and preparedness priorities include proactive outreach, chronic-care continuity, rapid medical assistance, and targeted transport, shelter, cooling, and medicine support for susceptible populations. These priorities follow social-vulnerability evidence and the need to prioritize vulnerable communities in heat mitigation (16, 17).

Large adaptive-capacity-deficit profile. Potential monitoring and preparedness priorities include targeted intergovernmental transfers, drainage and green or cooling infrastructure, resilient primary-care facilities, workforce support, and sustained preparedness investment. Green infrastructure can provide important but unequally distributed cooling benefits (15), while resilient-health-system frameworks emphasize infrastructure, workforce, and continuity capacity (45, 46).

Compound-risk profile. When high hazard, dense exposure, social vulnerability, and adaptive deficits coincide, isolated payer responses may be insufficient; this follows the interaction-based IPCC risk framework and is illustrated by high-exposure, capacity-constrained settings (1, 28). Joint fiscal, insurance, provider, and household monitoring could inform coordinated deployment before events, rapid support during disruptions, and post-event evaluation of utilization and payment shifts, consistent with integrated health-system resilience planning (46). Specific allocations still require published factor scores, uncertainty assessment, claims evidence, and causal validation.

### 5.3 Limitations and future research

#### 5.3.1 Endogeneity and limits of causal inference

Endogeneity is the principal inferential limitation. Province and year fixed effects absorb time-invariant characteristics and common national shocks but not time-varying policy, adaptation investment, disaster transfers, insurance reform, hospital expansion, regulation, migration, or healthcare access (23). Reverse causality and anticipation are plausible because spending may build capacity that later reduces vulnerability, and budgets may rise before expected events. One-year lags do not resolve persistent confounding, serial dependence, anticipatory behavior, or joint evolution of expenditure and adaptation; the robustness checks are specification tests, not an identification strategy.

Future causal research should exploit defensible exogenous variation and observe the sequence from climate exposure to service use, claims, and payment. Options include event-study or difference-in-differences designs around unexpected events; staggered resilience, warning-system, flood-control, or insurance reforms with credible pre-trends; regression discontinuities at response or transfer thresholds; synthetic controls for major disasters; and instrumental variables based on atmospheric or upwind weather variation only when exclusion restrictions are scientifically justified. Dynamic-panel methods may address persistence but still require valid instruments and specification tests; these design requirements are consistent with standard panel-data identification principles (23).

#### 5.3.2 Outcome measurement and price deflation

Annual growth rates provide a common measure across payers but are sensitive to denominator effects, mean reversion, settlement delays, and short-term accounting changes. A high expenditure level raises the next year's comparison base, while delayed reimbursement or fiscal booking can separate service use from recorded payment. Log-level and lagged models probe but do not eliminate these problems. The coefficients therefore describe associations with annual payer-specific growth, not welfare loss, fiscal stress, or persistent financing burden.

Real expenditure was deflated using overall provincial CPI rather than a healthcare-specific index. Faster growth in medical-service, pharmaceutical, or insurance-payment prices would overstate real growth, whereas slower growth would understate it. Bias may differ across payers because public procurement, negotiated payments, and household retail prices follow different trajectories. Future replication should use medical-care CPI, pharmaceutical indices, or service-specific deflators where available and report plausible ranges under alternative price assumptions.

Province-level CPI also serves two roles: it constructs deflated outcomes and enters the model as a time-varying covariate. The covariate was intended to capture residual general-price variation, but this overlap may create mechanical dependence, absorb the same price signal twice, and complicate interpretation through over-adjustment or endogeneity. Fixed effects do not fully eliminate this concern. The CPI coefficient is therefore not interpreted structurally; future analyses should compare models that omit or lag CPI and those using healthcare-specific deflators or price components.

#### 5.3.3 Measurement-model and SEM limitations

EFA, CFA, and SEM used repeated province-year observations, and conventional fit statistics do not fully address clustering, temporal dependence, or fixed heterogeneity. Bootstrap-based goodness-of-fit assessment can supplement conventional SEM diagnostics (47), but it would not remove these panel-data limitations. Four factors were fixed and Varimax rotation imposed a theory-guided orthogonal simple structure rather than estimating a data-driven factor count or substantive independence, following standard multivariate-analysis practice (21). Although both the Fornell–Larcker and HTMT criteria were satisfied within the study sample, they were derived from the same repeated province-year observations used for EFA and CFA and therefore remain internal diagnostics rather than independent external validation. Future work should compare competing structures, use temporally or geographically separated validation samples, and assess longitudinal measurement invariance and stability across periods and regions. SEM lacks the fixed effects, covariate structure, and clustered inference of the primary models and remains descriptive.

CFA scores are generated regressors. Although they bridge the measurement and panel models, second-stage clustered standard errors do not fully propagate factor-estimation and measurement uncertainty. Bootstrap procedures that repeat score construction and panel estimation, plausible-value approaches, or one-step multilevel structural models would improve uncertainty quantification. The additive specification also does not estimate synergistic interactions or whether one dimension conditions another; compound-risk models require larger samples, prespecified functional forms, and careful interpretation.

Separate payer regressions identify payer-specific associations but do not test coefficient equality across government, social, and OOP outcomes. Different outcome variances and correlated disturbances make visual comparison insufficient. Future studies should use stacked equations, multivariate panel models, or seemingly unrelated specifications with common scaling and cross-equation Wald tests before claiming payer heterogeneity.

#### 5.3.4 Spatial aggregation, payer detail, and future data

Provincial aggregates cannot identify heterogeneity at city, county, institution, or individual level, and ecological associations do not show that the most exposed populations generate the observed expenditure. Associations may vary by age, income, disease, occupation, insurance type, benefit design, residential environment, and access to cooling, transport, or care. Future evidence should link high-resolution climate exposure to claims, utilization, medical-assistance records, and individual expenditure while preserving privacy and accounting for spatial spillovers.

The financing sources were not disaggregated by outpatient care, hospitalization, medicines, public health, chronic-disease management, emergency treatment, rehabilitation, or post-disaster prevention, and service timing could not be separated from insurer settlement or government accounting. Aggregate social financing should not be interpreted as basic medical insurance alone. A fuller pathway would connect event timing, diagnosis, service use, provider charges, reimbursement, settlement lags, public transfers, and household residual payments. Such data, combined with uncertainty-quantified risk profiles, are needed before the associations can guide payment design, fiscal transfers, or targeted household protection.

## Data Availability

The original contributions presented in the study are included in the article/supplementary material, further inquiries can be directed to the corresponding author.
